# Influence of patient demographics and socio‐economic status on treatment choices for permanent mature teeth with painful vital teeth: a pilot study in the Australian public dental system

**DOI:** 10.1111/adj.13069

**Published:** 2025-03-25

**Authors:** Y Alfaisal, OA Peters, G Idris, S Zafar, CI Peters

**Affiliations:** ^1^ School of Dentistry The University of Queensland Brisbane Queensland Australia; ^2^ Metro North Hospital and Health Services Queensland Health Brisbane Queensland Australia

**Keywords:** Treatment decisions, socio‐economic status, pulpitis, vital pulp therapy, root canal treatment, extraction, public dental service

## Abstract

**Introduction:**

Socio‐economic status influences treatment decisions. This influence remains uncovered in teeth with painful pulpitis.

**Aims:**

To investigate the influence of patients' demographics and socio‐economic status on treatment choices for permanent mature teeth with painful vital teeth.

**Methods:**

Records of adult patients who received extraction, root canal treatment and vital pulp therapy in public sector dental care were categorized. Correlation of patient age, gender and socio‐economic status with rendered treatments was investigated. Patients' socio‐economic status was determined using their postcode's Socio‐Economic Indices for Areas (SEIFA) scores; a high score indicates higher status. Three groups of n = 25 patients per treatment were randomly selected after applying the inclusion criteria. Data were analysed using chi‐square test, One‐way ANOVA and Kruskal–Wallis test.

**Results:**

There was no significant correlation between patient age or gender and treatment performed (*P* = 0.250, *P* = 0.683). SEIFA scores were higher for vital pulp therapy, then root canal treatments and lowest for extraction; however, no significant association existed between patients' socio‐economic status and treatment type (*P* = 0.210). A formal diagnosis was not documented in 8% of vital pulp therapies, 28% of root canal treatments, and 64% of extraction cases. Vital pulp therapy was never offered in root canal treatment or extraction groups. Pulp exposure guided vital pulp treatments, while patient preference drove half of root canal treatment and extraction choices.

**Conclusions:**

Patients age and gender did not affect treatment decisions. Socio‐economic status might influence treatment decisions in painful permanent teeth. The service setting appears to have a major impact.

**Abbreviations and acronyms**
CSCs: calcium silicate‐based materials
EPT: electric pulp testing
ISOH: Information System for Oral Health
MN‐OHC: Metro North–Oral Health Centre
RCT: root canal treatment
SEIFA: Socio‐Economic Indices for Areas
SES: socio‐economic status

## INTRODUCTION

Socio‐economic status (SES) has a significant effect on both general and oral health. For example, socio‐economic inequalities are associated with oral health status.^1^ Previous studies have showed a negative correlation between low socio‐economic position and oral health and dental disease.^2^ Additionally, it has been reported that low socio‐economic position might be associated with a higher risk of having carious lesions.^3^ According to Australia's National Oral Health Plan, people representing low socio‐economic groups continue to experience higher rates of dental disease when compared to individuals from more affluent backgrounds.^4^

Socio‐economic factors such as household income, education level and access to health care and insurance services can have significant implications on oral health status outcomes.^5^, ^6^ Additionally, these factors affect the decision‐making process regarding treatment provided to patients within the oral health sector.^7^ The SES of individuals could lead to patients receiving less than ideal or even non‐standard treatment as financial constraints often influence the decision‐making process.^8^ Moreover, limited financial resources might force both patients and practitioners to prioritize immediate symptom relief, such as extractions over more definitive or long‐term treatments such as RCT due to affordability concerns.^5^, ^8^ Overall, disparities in SES might contribute to inequalities in the delivery of oral health care. This trend is prominently observed in Australia, where profound social inequalities in health persist.^9^ It has been reported that individuals with low SES and those uninsured were disadvantaged in accessing dental care.^10^ Moreover, It has been reported that socio‐economically disadvantaged Australians who face barriers to accessing private dental care often suffer further oral health disadvantage due to the increased emphasis on extraction‐focussed services and inadequate emphasis on preventive and maintenance care in public clinics.^11^

The influence of SES on treatment decisions is of particular interest in cases of painful vital teeth where treatment options range from conservative approaches, such as pulpotomy, to more invasive interventions such as tooth extraction. Pulp‐ and tooth‐retaining procedures are preferable, as tooth loss can lead to subsequent changes in the dentofacial complex and negatively affect the quality of life of the patients.^12^, ^13^, ^14^ Extraction of teeth with irreversible pulpitis has been frequently reported in uninsured patients, low‐income patients and those with limited access to specialist care.^15^, ^16^

Root canal treatment (RCT) is often considered the preferred approach for mature permanent teeth with carious pulp exposure and irreversible pulpitis due to its high success rate (>90%),^17^, ^18^, ^19^ and its favourable prognosis.^20^ However, RCT, and in particular molar endodontics, is a complex and expensive procedure that requires a high level of training and clinical skills.^21^ On the other hand, VPT is a viable alternative to RCT for vital teeth with inflamed pulps.^22^, ^23^ Partial and full pulpotomy using calcium silicate‐based materials (CSCs) is reported to be successful treatment modalities for teeth with carious pulp exposure and symptoms indicative of irreversible pulpitis.^24^, ^25^, ^26^, ^27^ The advantages of VPT include preservation of vital pulp tissue facilitating continuation of root maturation, maintaining pulpal defence mechanisms against microbial ingress, reduced cost and shorter chair‐side procedural time.^28^ In addition, VPT offers retention outcomes comparable to conventional RCT in permanent teeth with pulpal diseases, further highlighting its effectiveness as a successful treatment modality.^29^, ^30^, ^31^

Understanding the relationship between SES and dental decision‐making is important for dental practitioners, specifically in delivering patient‐centred care and addressing disparities in oral health outcomes. This is particularly relevant when choosing between conservative and aggressive irreversible treatments, such as in the case of patients presenting with painful pulpitis.^32^ There is a clear evidence in the literature about the influence of patient and dentist‐related factors on treatment decision‐making^32^, ^33^; however, there is only scarce information about the impact of these factors, particularly SES, on treatment decisions in teeth with pulpitis.^32^

Most studies investigating decision‐making in dentistry used questionnaire‐based methodologies and were carried outwithin specific geographical settings. Although these studies are valuable, their adopted method provided limited evidence and might lack generalizability. It is therefore imperative to explore this area from a clinical perspective in well‐defined settings. Consequently, the current research was designed to investigate the impact of patient factors including SES on treatment decision‐making within the public dental service in Australia. For this pilot study, the largest oral health centre and teaching facility in Australia with the highest number of treated patients in the investigated area was chosen for this pilot investigation. A significant proportion of patients seeking treatment at this facility for painful teeth could potentially receive VPT as a treatment option.

## AIMS OF THIS STUDY

To investigate the influence of patients' demographics and SES on treatment choices for permanent mature teeth with painful pulpitis. The null hypothesis was that treatment decisions for these patients are not related to a patients' SES or demographics.

The specific objectives of this pilot study were as follows:

- to assess the quality of data related to treatment decisions for vital, painful teeth within a public dental setting in Australia;
- to investigate the impact of patient demographics, particularly SES, on the selected treatment approach in vital painful teeth; and
- to explore the differences in the number of previously extracted teeth between the treatments performed.

## MATERIALS AND METHODS

### Ethical considerations

Ethics approvals have been obtained from The University of Queensland (2022/HE001136) and Metro North Hospital and Health Services (HREC/2022/QPCH/80356).

### Study design and setting

This is a retrospective cross‐sectional clinical study, which collected data from patients' electronic records. These patients received dental care at the Metro North–Oral Health Centre (MN‐OHC) between December 2017 and December 2019. Matched case numbers of patients seen in a public dental clinic associated with a dental school were analysed.

### Study cohort

**Fig. 1:**
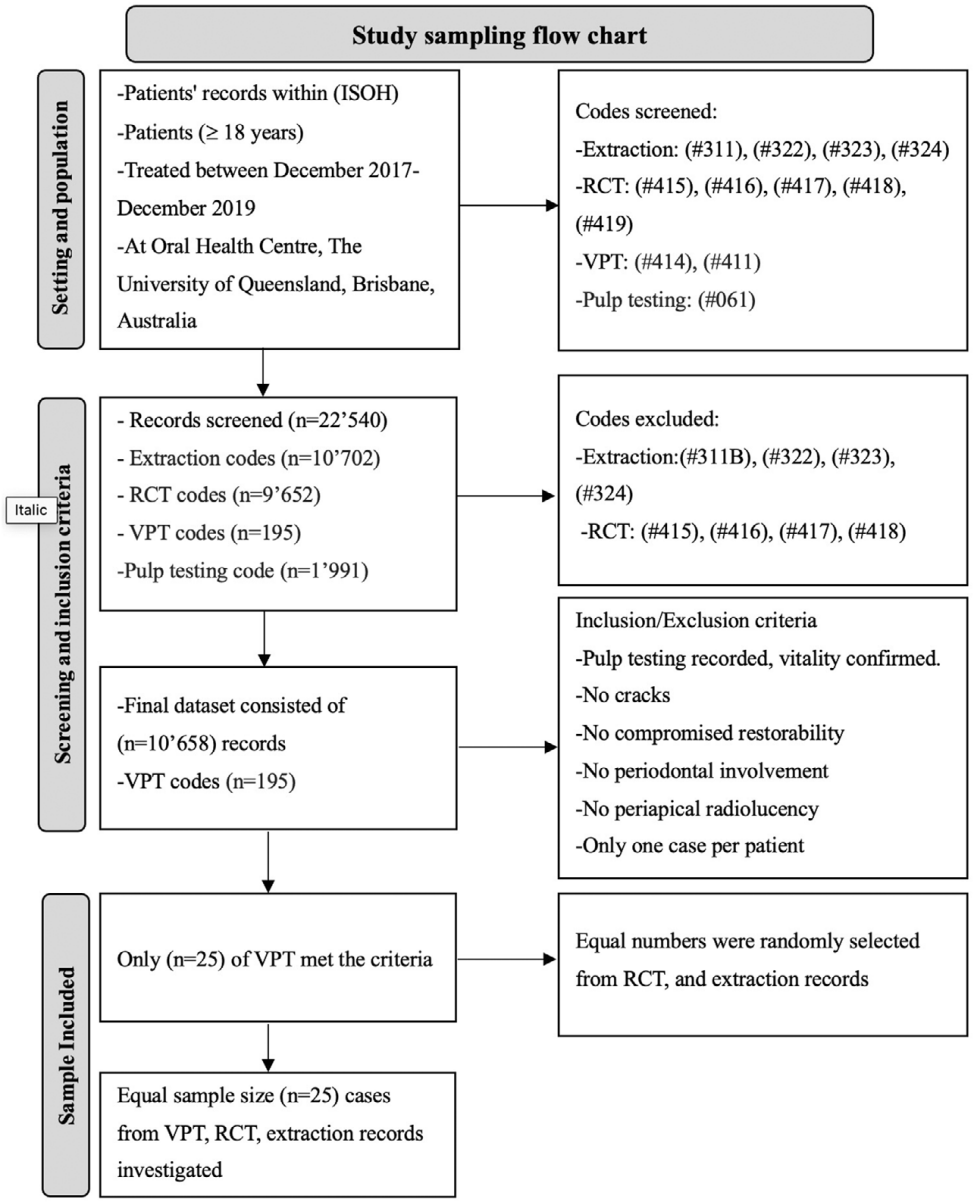
Study flow chart (selecting the sample size included in the analysis).

Records of patients aged 18 years or over were screened and reviewed in the patient's electronic records from Metro North's Information System for Oral Health (ISOH). Fig. 1 shows the pathway to the subset of data that were selected to be included in this study. These records were classified according to the treatment provided into extraction cases, RCT and VPT including pulpotomy and DPC. Australian Dental Association (ADA) item codes were used in the electronic records search as given in Table 1.

**Table 1:** Item codes used for the data extraction. The item codes are the recommended item code system by Australian Dental Association (ADA)

Item code	Details
Pulp testing	
061	Pulp testing—per appointment
Extraction	
311A	Removal of a tooth or part(s) thereof—1st tooth extracted from each quadrant
311B	Removal of a tooth or part(s) thereof—each subsequent tooth in same quadrant
322	Surgical removal of a tooth or tooth fragment not requiring removal of bone or tooth division
323 A&B	Surgical removal of a tooth or tooth fragment requiring removal of bone‐ 1st tooth and each subsequent tooth extraction
324	Surgical removal of a tooth or tooth fragment requiring bone removal and/or tooth division
Root canal treatment	
415	Complete chemo‐mechanical preparation of root canal—one canal
416	Complete chemo‐mechanical preparation of root canal—each additional canal
417	Root canal obturation—one canal
418	Root canal obturation—each additional canal
419	Extirpation of pulp or debridement of root canal(s)
Vital pulp therapy	
414	Pulpotomy
411	Direct pulp capping

To facilitate the search for the required cases, non‐relevant codes were eliminated so that only the following item codes remained: #061, #411, #414, #419, and #311A (see shaded items in Table 1).

### Data extraction and analysis

Initially, a total of 22 540 electronic patient records were screened, which were subsequently filtered and classified using electronic item codes for the following groups: extraction (n = 10 702); RCT (n = 9652); VPT (n = 195) and (1991) records of pulp testing with documentation of the respective item codes.

Among the 22 540 patient records, only 195 records included VPT item codes. Therefore, a final sample size of n = 195 cases was selected for each of the three treatment groups under investigation.

Then, the matched control cohorts from both RCT and extraction groups, with n = 195 records from each, were randomly selected respectively. In an initial analysis, matching groups of cases were compared based on demographic variables including age, gender distribution and SES, as described by SEIFA rank (Fig. 1).

A next elimination step resulted in n =10 658 records to be further queried for documentation of pulp testing (Table 1; Fig. 1). It was observed that out of all 195 VPT cases, pulp sensibility testing (item code #061) was only charged out in 25 cases. Accordingly, similar subsets of 25 cases from each treatment group (RCT and extraction) were randomly selected that fulfilled the inclusion criteria described (Fig. 1).

To generate cohorts of matched controls (n = 25 each) for RCT and extraction cases, respectively, the following allocation process was employed: from a randomized numbered list every 10th cases were examined for clinical findings that would have precluded the utilization of VPT. Specifically, sensibility testing by either cold testing or electric pulp testing (EPT) or both tests should be recorded, and pulp vitality confirmed. However, teeth with cracks, compromised restorability, advanced periodontal disease rendering the prognosis unfavourable, or those with periapical radiolucency were excluded. Additionally, only one tooth per patient was included (Fig. 1).

The final included patient records were reviewed, and the diagnostic information indicated that the teeth were reported as painful.

The following information was extracted: demographic data including postcode, age and gender. The number of missing teeth was tabulated as a surrogate measure for oral health status; additionally, details regarding sensibility tests, treatment procedures and treatment rationale were included. The inclusion of postcode information aimed to provide insight into the patients' SES.

Collected data were then exported on the Microsoft Excel (Microsoft Excel, Microsoft office 365, Microsoft Corporation, Washington USA) spreadsheet and anonymized with identifiers removed upon completion of the collection process. To ensure the confidentiality of patient details, no individually identifiable information was retained. The de‐identified data were cleaned and coded before being securely stored in the University of Queensland's (UQ) Research Data Manager (UQRDM).

Socio‐Economic Indices for Areas (SEIFA), specifically, the Index of Relative Socio‐economic Advantage and Disadvantage (IRSAD), was used to assess the patients' SES based on their postcodes. SEIFA combines census data such as income, education, employment, occupation, housing and family structure to provide a comprehensive overview of the socio‐economic characteristics of an area.^34^ This index (IRSAD) summarizes information about the economic and social conditions of people and households within an area. This index includes both relative advantage and disadvantage measures.^35^ A low score of IRSAD indicates a relatively greater disadvantage and a lack of advantage in general, whereas a high score indicates a relative lack of disadvantage and greater advantage in general. For ease of interpretation, it is recommended to use the index rankings and quantiles (deciles, percentiles) for analysis. In this study, ranks and percentiles were used to represent SEIFA scores to indicate a socioeconomic status. For ranks, areas are ranked in order of their score, from the lowest to the highest, with rank one representing the most disadvantaged area. In deciles, all areas are ordered from lowest to highest score; the lowest 10% of areas are given a decile number of one, the next lowest 10% of areas are given a decile number of two, and so on, up to the highest 10% of areas which are given a decile number of 10. This means that areas are divided into 10 equal‐sized groups, depending on their score. The same applies to percentiles, except that the areas are divided into one hundred equal‐sized groups, depending on their score; the lowest 1% of areas are given a percentile number of one, while the highest 1% of areas are given a percentile number of 100.^35^

### Statistical analysis

For the statistical analysis, the Jamovi software Version 2.3 was employed. Graphs were generated using GraphPad Prism for macOS, Version 10.0.2 (GraphPad Software, San Diego, CA, USA). The chi‐square test was used to evaluate associations between categorical data (gender) and treatment decisions. One‐way ANOVA was used to explore the association between patients' age and treatment performed. Moreover, Kruskal–Wallis test was utilized after confirming the non‐parametric nature of the data (SEIFA scores, number of missing teeth) through normality testing (Shapiro–Wilk test). The relationship between variables including patient's age, gender, number of extracted teeth and SEIFA scores, and the treatment procedures performed was further explored. The level of significance was set at *P* < 0.05.

## RESULTS

In the initial three groups of n = 195 (VPT, RCT and extraction), median (±95 CI), at the time of treatment the ages were 43 (40–46), 46 (43–48) and 48 (44–53) years for patients that received VPT, RCT and extraction, respectively. These differences were not significant (*P* = 0.0964, One‐Way ANOVA).

Regarding gender distribution, there were more women than men in both VPT (111 vs. 84) and RCT (109 vs. 86) groups, whereas the opposite gender distribution was noted in the extraction group (88 vs. 107) when treating vital painful teeth. This difference was statistically significant (*P* = 0.0355, chi‐square test), with both VPT and RCT groups being different from the extraction group. While there was a trend for the VPT group to score slightly higher in SEIFA ranks, this difference was not significant (*P* = 0.0547, Kruskal–Wallis test).

In the second stage of analysis, a detailed investigation was carried out on the records of VPT, RCT and extraction cases (n = 25 each), as shown in Tables 2, 3, 4. The gender distribution among patients was comparable in the VPT group; however, the proportion of women was slightly higher in the extraction group (56%) and notably highest in the RCT group (64%). This difference was not statistically significant (*P* = 0.683). The average age was 42.3 years among the VPT patients, 50.7 years among RCT patients and 47.8 years among the extraction group. Again, this difference was not statistically significant (*P* = 0.250) (Table 2).

**Table 2:** Demographic data for the three cohorts (n = 25 each)

	VPT	RCT	Extraction	*P* value
Age				
Mean	42.3	50.7	47.8	0.241*
Standard deviation	19.1	16.5	17.1	
Gender n (%)				
Male	12 (48%)	9 (36%)	11 (44%)	0.683**
Female	13 (52%)	16 (64%)	14 (56%)	

* ANOVA test (normally distributed data).

** Chi square test.

**Table 3:** Diagnosis and treatment information extracted from clinical records for the three cohorts (VPT, RCT, Extraction, n = 25 each)

	VPT	RCT	Extraction
	n (%)	n (%)	n (%)
Diagnostic tool			
Cold test	23 (92)	23 (92)	23 (92)
EPT	8 (32)	14 (56)	9 (36)
Percussion	22 (88)	21 (84)	16 (64)
Mobility	4 (16)	2 (8)	2 (8)
Palpation	5 (20)	8 (32)	3 (12)
Probing	3 (12)	0 (0)	0 (0)
Radiographs			
PA	21 (84)	25 (100)	19 (76)
BW	5 (20)	0 (0)	0 (0)
OPG	1 (4)	0 (0)	6 (24)
Diagnosis			
Not available	2 (8)	7 (28)	16 (64)
Pulpal	19 (76)	11 (44)	4 (16)
Pulpal and periapical	4 (16)	7 (28)	5 (20)
Options given to the patients			
Do nothing	10 (40)	12 (48)	4 (16)
Restoration	6 (24)	0 (0)	0 (0)
VPT	13 (52)	0 (0)	0 (0)
RCT	11 (44)	19 (76)	12 (48)
Exo	3 (12)	13 (52)	21 (84)
Treatment rationale			
Not available	3 (12)	13 (52)	7 (28)
Pulp exposure	15 (60)	0 (0)	0 (0)
Patient choice	4 (16)	12 (48)	13 (52)
Provider recommendation	0 (0)	0 (0)	5 (20)
Other*	3 (12)	0 (0)	0 (0)

* Other: affected dentine left, reversible pulpitis, wear.

**Table 4:** Relationship of number of missing teeth and SEIFA* (IRSAD) scores to treatment options using patients' postcodes extracted from clinical records

	VPT (n = 25)	RCT (n = 25)	Extraction (n = 25)	*P* value*
Oral health status (number of previously extracted/missing teeth)				
Median	1	0	1	0.385
Range	0–6	0–15	0–20	
Confidence interval	[0.702–2.34]	[0.636–4.00]	[1.25–5.47]	
SEIFA (IRSAD) percentiles				
Median	84.0	84.0	79.0	0.210
Range	21–99	21–99	4–97	
Confidence interval	[68.1–87.5]	[62.4–84.2]	[52.8–78.2]	
SEIFA (IRSAD) Deciles				
Median	9	9	8	0.291
Range	3–10	3–10	1–10	
Confidence interval	[7.22–9.18]	[6.69–8.83]	[5.77–8.23]	
SEIFA (IRSAD) Ranks				
Median	359	359	335	0.211
Range	89–422	88–420	17–411	
Confidence interval	[289–373]	[265–358]	[224–332]	

SEIFA, Socio‐Economic Indexes for Areas, (IRSAD) Index of Relative Socio‐economic Advantage and Disadvantage.

* Kruskal–Wallis test (nonparametric data).

Regarding the diagnostic tests employed, the cold test was the most utilized test for sensibility assessment across all three treatment groups (92%). EPT was employed in 32% of VPT cases, in 56% of RCT cases, and in 36% of extraction cases (Table 3). Periapical radiographs were exposed in most of the cases, with rates of 84% in the VPT group, 100% in the RCT group and 76% in the extraction cases. A formal pulpal diagnosis was documented in the treatment notes in 92% of the VPT cases, 72% of RCT cases, and only 36% of extraction cases. Other diagnostic tests were also included; see Table 3.

Interestingly, VPT was never documented as a suggested treatment option for patients who received either RCT or extraction. Moreover, pulp exposure emerged as the primary reason described for selecting VPT as a treatment, while patient preference was the rationale for the treatment in almost half of RCT and extraction treatments, as detailed in Table 3.

**Fig. 2:**
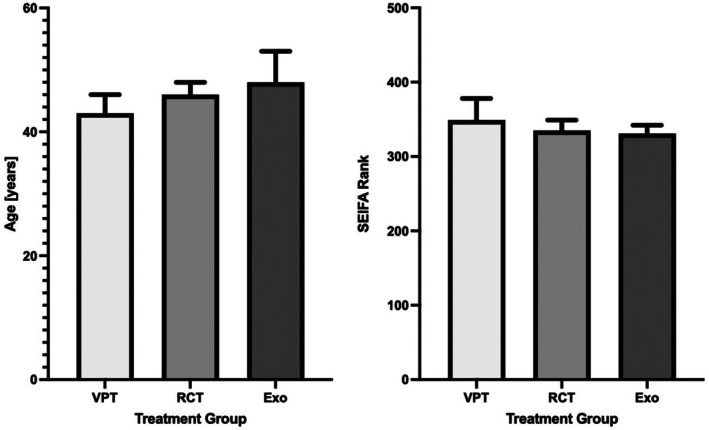
Bar charts showing medians and 95% confidence intervals for patient age and SEIFA rank, based on the initial groups (n = 195 each). SEIFA ranks for all included postcodes have a range of 427 to 1.

**Fig. 3:**
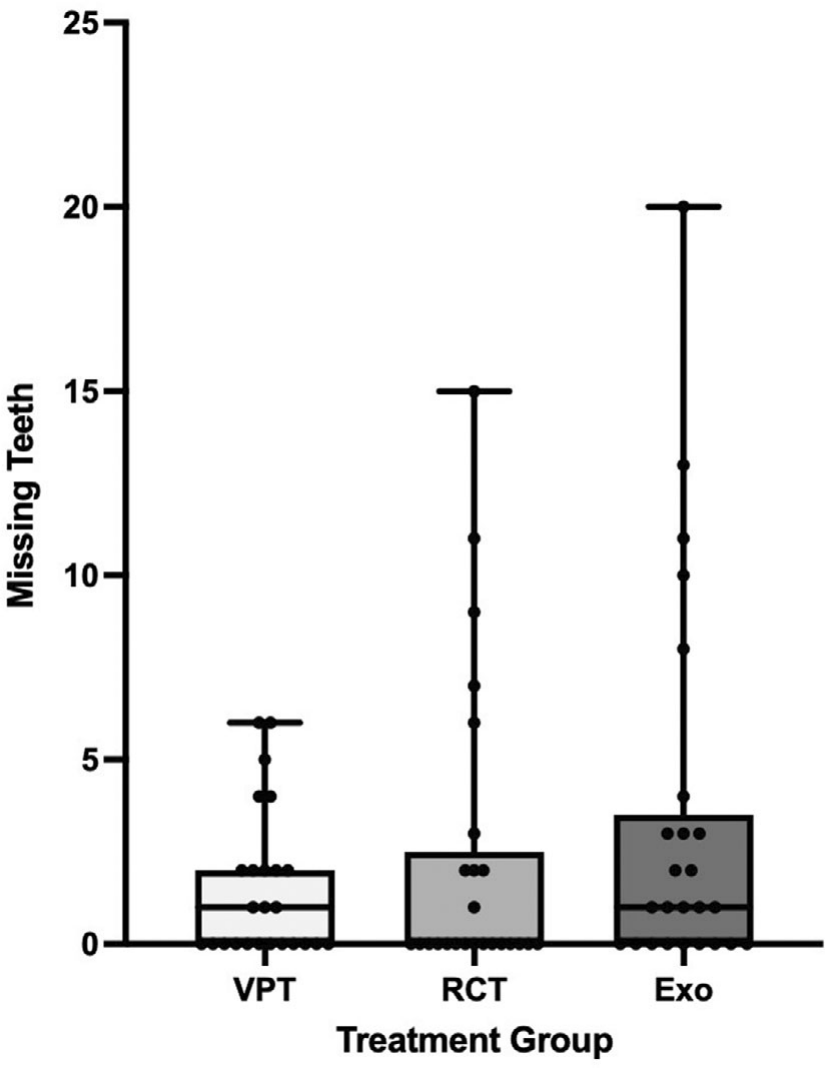
Boxplots with individual numbers of missing teeth at the treatment appointment illustrating the relationship between oral status and treatment offered for painful vital teeth.

The average number of missing teeth at the time of the provided treatment was lowest for the VPT group, with a mean of 1.52 teeth, followed by the RCT group, with a mean of 2.32, and highest for the extraction population, with a mean of 3.36 teeth (Fig. 2). However, considering that the data are not normally distributed, statistical analysis using the median showed an insignificant difference between the treatment groups (*P* = 0.385) (Table 4; Fig. 3).

**Fig. 4:**
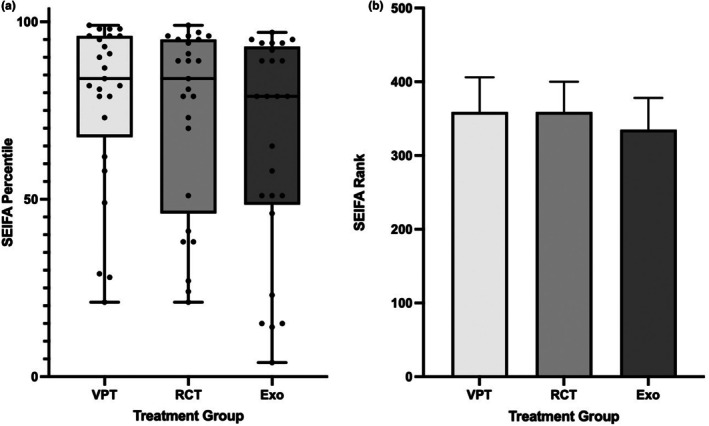
The association between SEIFA percentiles and ranks, and the treatment performed A is a boxplot with individual percentiles shown, while B shows the median and 95% Confidence intervals for SEIFA ranks. SEIFA scores are not normally distributed.

Regarding SEIFA/IRSAD scores expressed as percentiles, the average score for VPT was 77.8, 73.3 for RCT cases, and for extraction it was 65.5. There was a trend for SEIFA scores to be higher for VPT, followed by RCT and lowest for the extraction group (Fig. 3). However, there was no statistical significance associated with SEIFA percentile scores and treatment procedures performed (*P* = 0.210); the same applies to SEIFA deciles and ranks, as shown in Table 4 and Fig. 4.

The subsets of 25 cases each were statistically similar in age and SEIFA score distribution compared to the initial sets of 195 cases each (see Tables 2 and 4; Figs 2 and 4).

## DISCUSSION

This pilot study investigated the interplay of patient demographics and SES in determining treatment for painful permanent mature teeth with vital pulps. It focussed on two key objectives: to understand the data quality related to treatment decisions for vital painful teeth; to explore the differences in the number of previously extracted teeth between the treatments performed; and to study the impact of patient demographics and SES on the selected treatment approach in cases that could have potentially been treated with VPT.

When investigating treatments rendered and the influence of patient's SES on decision‐making in mature teeth with pulpitis, it became apparent that only a minimal fraction of the patient cohort did indeed receive VPT. We initially anticipated that providers at the clinic would follow currently available best evidence‐based protocols, as the clinic is associated with The University of Queensland's School of Dentistry. However, only a small proportion 195/22 540 included patients records had VPT treatment (pulpotomy, direct pulp capping) codes documented, while approximately 10 000 records each listed extraction and RCT codes. A mere 25 records of VPT cases met the inclusion criteria, where pulp testing was performed, and its code was documented. While the remaining VPT teeth might have had pulp testing done, the testing code was not recorded, and they were therefore excluded from the analysis. Therefore, the records of 25 patients in each treatment option (VPT, RCT, extraction) were investigated in detail.

While the null hypothesis confirmed that there was no impact of patient demographics such as age and gender on treatment decisions, a trend indicated that higher SES (SEIFA scores) was associated with VPT cases, followed by RCT and the lowest SES was associated with extraction treatment.

The subset groups of n = 25 cases each were statistically similar in age and SEIFA scores distribution compared to the initial sets of n = 195 cases. This indicates that the resultant outcomes from the smaller groups of n = 25 were representative of the sampled population for both SEIFA scores and age. However, the statistical analysis did not reveal a significant association between SES and treatment performed. This could possibly be attributed to the small sample size included in this pilot study.

Moreover, the study included patients' records from a single public sector location, which might have resulted in a relatively homogeneous SES distribution within the patient population, thus failing to represent the broader socio‐economic diversity within the community.

A similar trend was observed in the oral health status approximated by the number of missing teeth. It was noticed that this number was the least (i.e. the oral health status the best) for the VPT group, followed by the RCT group, and the highest in the extraction group. This finding does match what has been reported in the literature as limited financial means might lead to prioritising the immediate relief of symptoms such as through extractions over definitive or long‐term treatments such as RCT due to affordability concerns.^5^, ^8^ Moreover, it has been reported that socio‐economically disadvantaged Australians who receive dental care in the public sector suffer further oral health disadvantage, as public services have more emphasis on extraction of teeth and less emphasis on preventive and maintenance care.^11^

The influence of patient‐and dentist‐related factors on treatment decision‐making has been recently highlighted in the literature.^33^ However, there is a notable scarcity of information regarding the determinants shaping treatment decisions for teeth with pulpitis,^32^ with most of the studies relying primarily on questionnaire‐based methodologies.

The current study was designed to provide insights from a clinical perspective, offering a more objective approach to study factors influencing treatment choices,^36^ with a particular focus on patients' SES as a potential influencing factor on treatment decision‐making.

The present study showed prominent strengths, notably in its rigorous adherence to strict inclusion/ exclusion criteria; crucially, only cases with confirmed vitality through pulp testing were included. Additionally, it included only one case per patient to avoid clustering. The study presented a convenience sample, presenting a range of patients from various postcode addresses.

In the literature, SES has been consistently linked to differences in oral health outcomes. Studies revealed that individuals from lower income and education groups tend to experience higher burdens of untreated dental decay and overall poorer oral health.^3^, ^37^ This socioeconomic disparity might lead to a decrease in the oral health‐related quality of patient care and result in undesirable consequences.^8^ A recent study carried out in Queensland, Australia shed light on the association between area‐level SES and access to fluoridated water. Interestingly, it found that areas of greater socio‐economic disadvantage and with fewer economic resources are less likely to have access to fluoridated water, leading to implications for oral health‐related outcomes.^38^

Additionally, treatment options presented to the patient play a crucial role in choosing the management approach. Interestingly, in the current study, VPT was never offered to patients as a treatment choice in either the RCT or extraction groups. This could be attributed to the knowledge of dentists practicing in public sectors and the curriculum taught to undergrad students, as these cases were predominantly managed by undergraduates under the supervision of dental officers, adding further complexity to the decision‐making in pulpitis cases. Nevertheless, dentists' capacity to provide definitive pulpotomy might be constrained in publicly funded health services,^39^ potentially due to significant challenges associated with performing VPT in these settings, such as limited access to magnification and access to mineral trioxide aggregate (MTA) or calcium silicate cements (CSCs),^39^ which are considered to be a crucial factor in achieving predictable VPT outcomes.^26^, ^40^ Moreover, time constraints, available especially in emergency appointments, where most symptomatic pulpitis cases are presented, pose further obstacles to performing VPT effectively.

On the other hand, the option of doing nothing was occasionally presented in both populations. Furthermore, the diagnosis was mostly documented in VPT cases, less so in the RCT group, and the least in the extraction group. This discrepancy in diagnostic documentation might further explain the treatment options chosen, as determining the correct diagnosis is critical to providing appropriate management approaches.^41^, ^42^

Having access to a public data, including patients records, is considered a valuable resource to assess the trends and facts about preferred treatment options in certain conditions, such as irreversible pulpitis. This would provide strong evidence about the studied topics. However, based on the current study's findings, it is crucial to improve the database by conducting multiple audits to ensure consistent and accurate recording of treatment codes. This will help maintain the database as a reliable and valid resource for evaluating diagnostic protocols, treatment choices, and outcomes.

There were several limitations identified in the current study, such as the small sample size due to the limited number of records meeting the inclusion criteria. Additionally, the number of VPT cases was limited; however, there were quite possibly more cases of VPT within the reviewed records, but incomplete record keeping made it difficult to identify them. This highlights the importance of auditing the record‐keeping process, especially since most of these records are maintained by undergraduate students.

While a modest association between socioeconomic status and provision of VPT was observed, this association is likely to be confounded by other patient and dentist‐related factors. However, the electronic records lacked the necessary data to study these variables. In general, to better identify the factors influencing decision‐making, conducting a prospective study would allow for a more controlled investigation of these variables. This was not the case with the limitation of the current study, which was a retrospective review of the available records.

Moreover, this study included patient records from a single site, the Oral Health Centre, which might not fully represent the broader population, introducing potential bias associated with the patient population and the services provided in such a setting. Furthermore, a remarkable portion of the cases included in this study were treated by undergraduate students in the oral health centre, which might potentially impact the quality and consistency of the care provided.

## CONCLUSION

The findings of the current study highlighted a trend regarding the association between SES and the choice of treatment for pulpitis cases. Notably, higher SES was observed more frequently with VPT, followed by RCT, and the lowest for extraction cases; however, this was not statistically significant. Moreover, there was no significant correlation found between patients' age nor gender and the treatment procedure performed. The study also showed a notable lack of offering VPT as a treatment option in cases treated with RCT and extraction. Pulp exposure emerged as the most frequently cited reason available for choosing VPT as a treatment option for vital pulp cases.

## FUTURE DIRECTIONS

While this study hints at socio‐economic influence on treatment choices in permanent mature teeth with pulpitis, further research with a larger sample size and data from multicentre health care settings is required to validate the trends found in this study and to uncover any potential significant differences. The pilot nature of the current study makes it crucial to confirm the current findings with future prospective clinical studies and covering a wider range of dental practitioners and patients. Furthermore, the knowledge of the dentists in public sectors and the curriculum taught to undergrad students might require more focus on the importance of VPT as an option for teeth with pulpitis regardless of the SES of the patient. Developing a better understanding of the complex interplay between SES and dental decision‐making is essential for dental practitioners to deliver effective and equitable care and can promote better oral health outcomes and reduce disparities among diverse patient populations. Moreover, the challenges associated with performing VPT in public health services must be identified and addressed.

## AUTHOR CONTRIBUTIONS

**Y Alfaisal:** Methodology; validation; formal analysis; investigation; data curation; writing – original draft; visualization. **OA Peters:** Conceptualization; methodology; writing – review and editing; supervision. **G Idris:** Formal analysis; writing – review and editing. **S Zafar:** Writing – review and editing; supervision. **CI Peters:** Conceptualization; methodology; writing – review and editing; supervision; project administration.

## CONFLICT OF INTEREST

The authors declare that they have no conflict of interest.

## FUNDING INFORMATION

The authors declare that no funding was received for this study.

## PATIENT CONSENT

Patient consent was not necessary as the data were anonymous and retrospective as per ethics approval.

## Data Availability

The original data is available upon request.
